# Assessing the Perceived Value of Neuroethics Questions and Policy to Neuro-Entrepreneurs

**DOI:** 10.3389/fnins.2021.702019

**Published:** 2021-10-13

**Authors:** Ankita U. Moss, Zone R. Li, Karen S. Rommelfanger

**Affiliations:** ^1^Neuroethics and Neurotech Innovation Collaboratory, Neuroethics Program, Emory University Center for Ethics, Atlanta, GA, United States; ^2^Department of Neurology, School of Medicine, Emory University, Atlanta, GA, United States; ^3^Department of Psychiatry and Behavioral Sciences, School of Medicine, Emory University, Atlanta, GA, United States

**Keywords:** innovation, attitudes, venture capitalists, neuroscience, culture, business case for neuroethics, entrepreneurship, neurotechnology

## Abstract

Neuroscience and its findings have deep personal and cultural meaning, so the implications of brain science raise new flavors of ethical issues not covered by traditional bioethics. The field of neuroethics bridges this gap, addressing and responding to the ethical, legal, and social issues intimately related to the evolving landscape of neuroscience. Neuroethical concerns have registered at the highest levels of government. In 2018, an interdisciplinary global neuroethics group working with leading scientists from the International Brain Initiative, a consortium of seven large-scale national-level brain research projects around the globe, published “Neuroethics Questions to Guide Ethical Research in the International Brain Initiatives.” The document provides guiding questions to consider throughout the lifecycle of neuroscience research. These questions tackle issues such as identity, morality, cross-cultural differences, privacy, and potential stakeholder involvement in ethical decision-making. In our work with the International Brain Initiative, we noted the important role that the private sector will play in translating and scaling neuroscience for society. We also noticed a gap in communication and collaboration between government, academia and the private sector. These guiding questions were largely co-created with policy makers and academics, so it was unclear how these issues might be received by neuro-entrepreneurs and neuro-industry. We hoped to identify not only common concerns, but also a common language for discussing neuroethical issues with stakeholders outside of government and academia. We used empirical ethics methods to assess the perceived value and attitudes of neuro-entrepreneurs toward neuroethical issues and whether or not these issues align with the process of neuro-innovation. We conducted one-on-one structured interviews with 21 neuro-entrepreneurs in the private sector and used two independent reviewers to analyze for themes. From this preliminary research, we identified key neuroethical themes and processual pain points of neurotech entrepreneurs throughout the innovation process. We also provide a preliminary neuroethics needs assessment for neuro-industry and suggest avenues through which neuroethicists can work with neurotech leadership to build an ethically aligned future. Overall, we hope to raise awareness and provide actionable steps toward advancing and accelerating societally impactful neuroscience.

## Introduction

Neuroethics is a discipline that analyzes the “the social, legal, ethical and policy implications of advances in neuroscience” (International Neuroethics Society [INS], 2020). Neuroethics has responded to the rapid developments in neuroscience and has been developed alongside cutting-edge neuroscience. Issues of cognitive enhancement, loss of privacy, and identity are all present and future concerns of the field of neuroethics. Neurotechnologies, which have the possibility to augment or ‘enhance’ the brain or predict risk for diseases like Alzheimer’s, may alter future societal definitions and boundaries regarding what it means to have merit and what it means to thrive within a society (Farah, 2012; Ahlgrim et al., 2019).

The expansive implications for neuroscience have drawn attention and action from the highest levels of government. For example, neurotechnology, in the view of the transnational policy organization, the Organization for Economic Cooperation and Development (OECD), was the first emerging technology for which unique principles were deemed necessary (Organisation for Economic Co-operation and Development [OECD], 2019). The United States National Institutes of Health (NIH) BRAIN Initiative’s Neuroethics Working Group was created to navigate ethical issues and future implications in neuroscience research, such as challenges to autonomy and privacy unique to gathering and utilizing brain data (Greely et al., 2018). Such activities represent concerted efforts to maximize the positive impacts and minimize the possible negative impacts of emerging neurotechnology. Notably, these conversations were largely oriented toward and created by policymakers and the academic and government-funded research community. Robust neuroscience research and advancement has not only expanded internationally, but also has recently grown with entrepreneurship and the tech industry (Crunchbase, 2021). The 2020 current worldwide market for neurotechnology is estimated to be 11–14 billion US (Cavuoto, 2020). According to neuroscience market tracking entities like Sharpbrains, 70% of the patents in neurotech are in small and large industry (SharpBrains, 2020). The biggest market is in healthcare, with the second being for non-clinical (like wellness or fitness) use by consumers. An influx of neuroscience companies and “neuro-entrepreneurs” could promote access to neuroscience advances to markets across the globe from healthcare to consumer products.

While some management and entrepreneurship studies define “neuro-entrepreneurship” as the application of cognitive science and neuroscience to the practice of entrepreneurship itself, we refer to a “neuro-entrepreneur” in our study, as any individual who creates, deploys, or works on a neuroscience product within the private sector (Binder et al., 2015). Neuroscience products can range from brain-machine interfaces to pharmaceuticals to neuro-marketing tools and personality assessments utilized in the workplace. Such spheres can be broadly categorized under the umbrella term “neuro-industry,” an interdisciplinary sector that creates or deploys commercialized “neuro-innovation” or “neurotechnology.” Neuro-innovation and neurotechnology are conceptualized broadly and used interchangeably in the context of this paper and encompass novelty including hardware, software, pharmaceuticals and other brain-adjacent technologies. As neuro-industry rapidly grows, its implications for society must be considered. Neuro-industry does not solely consist of medical or health technology and can also consist of many non-clinical applications, which prompts the need for a careful risk-benefit ethics analysis for healthy individuals interfacing with these unprecedented brain technologies.

In 2018, an interdisciplinary global neuroethics group working with leading scientists and ethicists from the International Brain Initiative, a consortium of seven large-scale national-level brain research projects around the globe, published “Neuroethics Questions to Guide Ethical Research in the International Brain Initiatives,” or “NeQN” for short (Box 1). The NeQN outline essential questions to guide neuroethical inquiry and advance neuroscience research related to how neuroscience may raise issues with identity, morality, cross-cultural differences, privacy, and potential stakeholder involvement in ethical decision-making (GNS Delegates et al., 2018). In our work and global policy conversations, we have heard how important the private sector is in translating and scaling neuroscience for society, yet there is a significant gap in communication and collaboration across government, academia and the private sectors. It is also unclear how the NeQN might be received by neuro-entrepreneurs and neuro-industry. Do the values, goals, and processes of most neuro-entrepreneurs and a relatively young neuro-industry community align with integrating neuroethics questions like the NeQN into their processes of innovation?

**Box 1. Neuroethics questions to guide ethical research in the international brain initiatives: NeQN (from GNS Delegates et al., 2018).**

**Q1. What is the potential impact of a biological model or neuroscientific account of disease on individuals, communities, and society?**

1a. What are the possible unintended consequences of neuroscience research on social stigma and self-stigma?

1b. Is it possible that social or cultural bias has been introduced in research design or in the interpretation of scientific results?

**Q2. What are the ethical standards of biological material and data collection and how do local standards compare to those of global collaborators?**

2a. How can human brain data (e.g., images, neural recordings, etc.), and the privacy of participants from whom data is acquired, be protected in case of immediate or legacy use beyond the experiment?

2b. Should special regard be given to the brain tissue and its donors due to the origin of the tissue and its past?

**Q3. What is the moral significance of neural systems that are under development in neuroscience research laboratories?**

3a. What is the requisite or even minimum features of *engineered* neural circuitry required to generate a concern about moral significance?

3b. Are the ethical standards for research conduct adequate and appropriate for the evolving methodologies and brain models?

**Q4. How could brain interventions impact or reduce autonomy?**

4a. What measures can be in place to ensure optimal autonomy and agency for participants/users?

4b. Who will have responsibility for effects (where responsibility has broad meaning encompassing legal, economic, and social contexts)?

**Q5. In which contexts might a technology/innovation might be used/deployed?**

5a. Which applications might be considered misuse or best uses beyond the laboratory?

5b. Does this research raise different and unique equity concerns and, if so, have equitable access and benefit of stakeholders been considered?

This project explores the value of neuroethics to neuro-entrepreneurs and neuro-industry leaders by exploring how neuroethics might be aligned with the creative process of neuro-entrepreneurs and neuro-industry. Information on the sociology of innovators, and particularly neuro-entrepreneurs, is integral to determining whether neuroethical guidelines could serve neuro-entrepreneurs whose work may have implications for a global stage, and thus, society at large. In order to assess the value of neuroethics to neuro-entrepreneurs, and the relationship between ethics and innovation generally for entrepreneurs and innovators, we conducted a series of one-on-one interviews with neuro-entrepreneurs investigating whether or not ethics and innovation are viewed as compatible, mutually exclusive, or perhaps somewhere in between.

## Materials and Methods

### Rationale for Qualitative Research and Empirical Neuroethics

We conducted an empirical neuroethics study consistent with and draws from the standard methodologies in Qualitative Research Design and Grounded Theory (Ragin et al., 2004; Ragin and Amoroso, 2011). Ethics is gaining increasing momentum to establish empirically driven work (Goldenberg, 2005; Frith, 2010; Strech, 2010) and draws from established social science mixed-methods (i.e., mixing qualitative interviews and quantitative surveys) (Creswell, 2009; Babbie, 2010; Ragin and Amoroso, 2011). These studies are designed to gain breadth and richness of perspectives on some common neuroethics topics rather than a generalizable, decisive definitions of ethics, for example. Our goal was to attempt to gather information to analyze the interviewees perspectives. Their words and personal descriptions that were relevant to their respective contexts as entrepreneurs in neuroscience. As we look to bridge gaps in communication, language and understanding between the public and private sector, offering representative quotes from neuro-entrepreneurs can be an important starting point for conversations to bring diverse stakeholders into the room together.

### Interviews and Data Collection

Semi-structured interviews were used to collect data regarding attitudes toward neuroethics, while allowing for follow-up questions to probe for clarity and more nuanced individual responses and perspectives. The conceptual map and NeQN together provided the framework for the initial iteration of the interview guide. The interview guide was oriented to solicit perspectives on the purpose of neuro-innovation and views on five neuroethical themes (GNS Delegates et al., 2018).

*These are simplified as follows:*

1. The impact of brain disease models and stigma.
2. The ethical standards of biological/neural data collection and privacy.
3. The moral significance of neural systems.
4. The impact on or challenges to individual autonomy.
5. Appropriate contexts for neuro-innovation usage and deployment and diverse stakeholder involvement.

Qualitative data collection via comprehensive interviews was conducted with 21 neuro-entrepreneurs consistent with standard methods in qualitative research (Ragin et al., 2004). The cohort of these 21 neuro-entrepreneurs represented industry in the United States, Europe, and Australia (Supplementary Appendix 1). Participant data collection ended at 21 entrepreneurs when we confirmed we had met our goal of exhausting themes, and repeatedly no new themes emerged.

After obtaining verbal consent from the participants, one-on-one semi-structured interviews (the interview questions guided the conversation, which honed in on specific topics relevant to the biography of the neuro-entrepreneur for depth), lasting from 30 to 60 min, were conducted. These interviews helped gauge attitudes toward neuroethics within neuro-industry, as well as explored perspectives on the purpose and process of innovation itself. Interview conversations were recorded and transcribed in order to preserve the true syntax and meaning of the content. All participants were made aware of the recording and verbally consented to participate in the interview and data collection process. All recordings and identifying information were stored on password-protected devices. All interviews were recorded on the interviewer’s password-protected Zoom account. Throughout the process of data collection and data integration into this final work, all digital files were given pseudonyms and codes to protect the identity of the participant. Participants were made aware of the protection measures and agreed to continue with the data collection process with the knowledge of a possible and unanticipated breach in confidentiality.

After the recording process, the interviews were transcribed through a transcription service. After the interview had been transcribed, stored on a password-protected account on Emory University Box (secure storage server), and analyzed, the audio file was destroyed. All identifying information was stored on the password-protected devices on secure servers.

Recruitment was via email. The researcher’s email is password-protected. Only the research team had access to the password-protected emails and subsequent documents that included identifiers linking codes to participants. Interview times were mutually agreed upon. All interviews were performed remotely. At the end of the interviews, the researcher asked the participants if they could be contacted in the future should the study need clarification of information collected during the interview.

### Conceptual Map and Thematic Analysis

**FIGURE 1:**
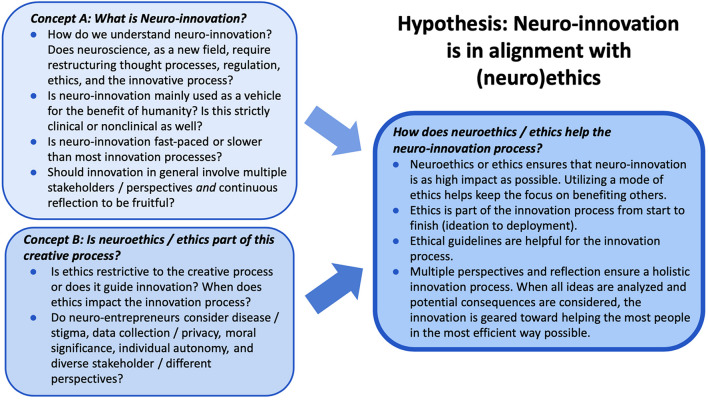
Concept A: What is Neuro-innovation? Concept B: Is neuroethics/ethics part of this creative process? Concept C: How does neuroethics/ethics help the neuro-innovation process? We formulated the hypothesis that aligning the motivations and purpose of innovation with specific neuroethics questions that arose during the innovation process could help point to specific areas where neuroethics could assist and advance the neuro-innovation process.

Before the creation of the interview guide, the research team formulated a preliminary conceptual map that integrated the concepts of innovation and ethics and demonstrated our thinking on how the concepts could be related and then categorized. Innovation (concept A) and ethics (concept B) gradually grew to the questions “What is Neuro-Innovation?” (innovating within the sphere of neuroscience) and “Is neuroethics/ethics part of the creative process?” respectively. Under each preliminary concept, are key terms and phrases that could possibly represent a certain identified theme during the interview data collection process. Concepts A and B come together in the resolution of C “How does neuroethics/ethics help the neuro-innovation process?” component of the conceptual map (Figure 1).

We utilized a standard grounded theory analytical approach to coding and thematic analysis, which allowed for reiterations and re-integrations of concepts A and B after each interview (Weiss, 1994). We utilized deductive analysis to explore dimensions as related to the NeQN and our specific neuroethics questions and also utilized inductive analysis to explore the emergence of new insights that were not anticipated. Themes were analyzed through an iterative process by two independent coders. Through constant comparative analysis, each interview was examined and compared to prior interview data to create finer-grained conceptual categories and themes, leading to a comprehensive and consistent theory and coding scheme (i.e., a codebook of categories and analytic strategy). Throughout data collection, memos in data analysis and categorization were captured. Iterations of the conceptual map were created in tandem with the collective responses of the neuro-entrepreneurs unfolded throughout the analysis (as is protocol in grounded theory). We refined our specification for categories and stopped interviewing when we had exhausted the emergence of new themes.

In order to ensure that annotations matched the attitudes of the neuro-entrepreneur at the time and that tone was preserved, the research team created a password-protected document with reflections that were written after each interview. These data, the analysis, and categorization of receptiveness and perceived values of neuroethics continuously informed the hypothesis and conceptual map as well. Throughout the analysis, the hypothesis that neuro-innovation could be aligned with neuroethics was solidified as seen in the final concept map.

## Results

The following are the final themes identified at the end of the data collection process (at the end of the 21 interviews). The theme categorization (Table 1) is derived from the final version of the conceptual map (Figure 1), which was informed by the Cultural Swirl Theory (Welz, 2003) and also explored neuroethical categories highlighted by the NeQN (GNS Delegates et al., 2018).

**TABLE 1:** Themes.

**A. What is the purpose of neuro-innovation(s)?**
1. Benefiting/advancing humanity: Reducing suffering from disease and injury to lack of access and ability and increasing happiness.
2. Clinical: Neurotechnology can address unmet clinical needs, improve treatment, and provide extra diagnostic accuracy/prediction, impacting how diseases are labeled, defined, and treated.
3. Non-clinical: Non-clinical development of neuro-innovation is inevitable and follows the course/cycle of innovation. Specifically, neuro-innovation will move from the clinical to non-clinical space.
4. Empowerment: Neurotechnology/neuro-innovation should aim to help people and enhance “autonomy” of the public, empowering them to have greater breadth of knowledge, choices, and behaviors.
**B. What are the key (neuro)ethical tensions of neuro-entrepreneurs?**
1. Data ownership: Users should own their data, but the business model doesn’t allow for it (Small companies are more incentivized to sell data for growth).
2. 2 Access and justice: Neuro-innovations at their best can empower society, but the tech and insights are not always shared with everyone in society who might benefit.
3. Neurodata and misuse: Current data regulations suffice but may not be sufficient for future implications and possible uses of brain data especially in the commercial space.
4. Shifting societal norms: Unintended uses or access to data and tech may lead to stigma, discrimination, power imbalances, and other unintended consequences, but the implications are not usually apparent to users or the entrepreneurs.
5. Autonomy: Neurotechnology/neuro-innovation can enhance or diminish autonomy.
**C. How would ethics fit with the neuro-innovation/creative process?**
1. Ensuring/maximizing impact: Ethics is desired for preventing harm to the end-user and ensuring impact.
2. Nimble guidance vs. restriction: Ethics enforcement is viewed as restrictive and slowing, primarily through the lens of regulation; however, ethical guidelines (in any format) can be helpful tools throughout and after the innovation process.
3. Incentivization: Incentivization of ethical behavior is missing and desired.
4. ROI (return on investment) and growth opportunities: Ethics could help neuro-entrepreneurs to maximize the uses of their products while also mitigating negative uses.

### Evidence of Themes

#### I. What Is the Purpose of Neuro-Innovation(s)?

**Table:** 

A. What is the purpose of neuro-innovation(s)?
1. Benefiting/advancing humanity: Reducing suffering from disease and injury to lack of access and ability and increasing happiness.
2. Clinical: Neurotechnology can address unmet clinical needs, improve treatment, and provide extra diagnostic accuracy/prediction, impacting how diseases are labeled, defined, and treated.
3. Non-clinical: Non-clinical development of neuro-innovation is inevitable and follows the course/cycle of innovation. Specifically, neuro-innovation will move from the clinical to non-clinical space.
4. Empowerment: Neurotechnology/neuro-innovation should aim to help people and enhance “autonomy” of the public, empowering them to have greater breadth of knowledge, choices, and behaviors.

1. Benefiting/advancing humanity: Reducing suffering from disease and injury to lack of access and ability and increasing happiness drives many neuroentrepreneurs.

Participants consistently noted an obligation to pursue innovations in neuroscience to broadly increase happiness and alleviate human suffering. Participants acknowledged that this goal to advance humanity and alleviate suffering needed to be met with consideration of the broad importance of ethical responsibility.

“I think that we’ve evolved a lot and there’s no need for suffering. And that being said, we have to find ways to do this kind of innovation in a responsible way, in an ethical way.” (X8)

Others were motivated by advancing the future of humanity and making the world a better place.

“So, I’m motivated by multiple factors. One is just the sheer fun of producing things and the other one is to **make the world better for people** inhabiting it.” (M21)

“I view neuroscience and neurotechnology as being the **entry point into a new future for humanity.** The brain is our final challenge of understanding ourselves and our relationship in the cosmos, and I think that technology not only can repair the brain and disease states and ameliorate the condition of patients with brain or nervous system disorders, but it can also illuminate a way forward for a greater understanding of what it means to be human. And that’s what excites me about this field.” (L12)

While some concerns were around alleviating disease/injury states, other motivations were around increasing broader happiness with access to increased knowledge of the self through brain technology and neuroscience knowledge. Part of this is also captured by thematic point 4 below on “Empowerment”.

2. Clinical: Neurotechnology can address unmet clinical needs, improve treatment, and provide extra diagnostic accuracy/prediction, impacting how diseases are labeled, defined, and treated.

“I think I see that **there’s a large unmet need of people with a range of neurological disorders and disabilities** that may not be curable or treatable with medicine, and bioelectronics or biomedical implants have the potential to help these people out.” (P19)

Participants noted that neuro-innovation is helpful for clinicians and medicine at large; neuro-innovation complements the diagnostic process and increases efficiency. Integrating neurotechnology into the clinical setting impacts the way diseases are labeled, defined, and treated. Participants also noted that neurotechnology will influence how diseases are diagnosed and perceived in society. Generally, neuro-innovations for diagnostic labels and predictive purpose (as with detecting risk for developing a disease prior to its ability to be diagnosed) were seen to have primarily positive implications.

“I think technology does play a role in what is disease and what is normal. I do think it does constitute a treatment…it can be used for selecting the right treatment.” (L1)

In the statement above, the neuro-entrepreneur states that neurotechnology itself can be a standalone treatment and can also complement the diagnostic process. Many neuro-entrepreneurs also noted that new neurotechnologies should complement rather than replace existing diagnostic and treatment strategies. However, many entrepreneurs voiced more wariness of non-clinical applications of neurotechnology.

3. Non-clinical: Non-clinical development of neuro-innovation is inevitable and follows the course/cycle of innovation. Specifically, neuro-innovation will move from the clinical to non-clinical space.

Off-label use of neurotechnology that moves into or is created for the commercial domain raised ethical concerns for the participants. The neuro-entrepreneur in the quote below states that labeling and categories that stem from neurotechnology and collected user data can have negative consequences for the individual and society.

“So, what we have to think about is, **something as benign as a fitness tracker could have unintended consequences**.” (U5)

Participants noted that neurotechnology that is deployed for commercial use is susceptible to exploitation upon its arrival to the market and that there are inevitable unknowns for the trajectory of technology use.

“I think sustainable growth and making sure that technology serves the common good and results in a **positive outcome for more people is what we should be aspiring toward**. But **I don’t think that that’s necessarily the case**.” (V14)

“I think it’s inevitable that someone will have a use case that’s unpredicted.” (J15)

The neuro-entrepreneurs above predict that commercial neurotechnology use cases will be variable and will sometimes result in unintended consequences rather than serving a common good.

4. Empowerment: Neurotechnology/neuro-innovation should aim to help people and enhance “autonomy” of the public, empowering them to have greater breadth of knowledge, choices, and behaviors.

Participants noted that neuro-entrepreneurs should aim to create innovations that empower individuals in multiple ways. This point is similar to the first thematic point of benefiting humanity and reducing suffering but is instead oriented more around individual benefit rather than broad societal benefit. The first example is around the notion of “restoration” or empowerment by healing.

“Well, I think that BCI will have a **massive impact on user autonomy for patients** that are paralyzed, particularly patients whose degree of paralysis limits their communication. So I would say that, I mean, when I think of BCI, I think of it as a restorative technology. And so essentially **allowing patients who have had some degree of autonomy** taken from them to return to as close to normal function as possible. So I think certainly it will restore autonomy for thousands, likely millions of people.”(O13)

Another entrepreneur explains how neurotechnology opens a new avenue of choices and knowledge for the individual to access.

“So, without any brain tech, I think right now, **many people feel they have very little autonomy because we are at the mercy of ignorance about the brain**, of ignoring the brain until it becomes a big clinical problem and then we are at the mercy of technicians, the psychologies, the psychiatrists and neurologists. So, I think, right now brain technology, both when you it is used by consumers directly or by professionals, many people are seeing that it promotes autonomy because it empowers people to start to make decisions and to be aware about something that, until now, they have had zero inside info.” (C2).

Increased development and deployment of neurotechnology for the enhanced autonomy of the user could deliver answers about the breadth and depth of neuroscience, while enhancing the human experience. The participants noted that neurotechnology should be created to enhance the human experience and expand individual capabilities in a positive way, and ultimately enhance autonomy in some way.

#### II. What Are the Key (Neuro)Ethical Tensions of Neuro-Entrepreneurs?

**Table:** 

B. What are the key (neuro)ethical tensions of neuro-entrepreneurs?
1. Data ownership: Users should own their data, but the business model doesn’t allow for it (Small companies are more incentivized to sell data for growth).
2. Access and justice: Neuro-innovations at their best can empower society, but the tech and insights are not always shared with everyone in society who might benefit.
3. Neurodata and misuse: Current data regulations suffice but may not be sufficient for future implications and possible uses of brain data especially in the commercial space.
4. Shifting societal norms: Unintended uses or access to data and tech may lead to stigma, discrimination, power imbalances, and other unintended consequences, but the implications are not usually apparent to users or the entrepreneurs.
5. Autonomy: Neurotechnology/neuro-innovation can enhance or diminish autonomy.

1. Data ownership: Users should own their data, but the business model doesn’t allow for it (Small companies are more incentivized to sell data for growth).

“**It’s easier to sell data than it is to preserve it and not sell data**. And I think increasingly we’re seeing pressure to sell data, that’s what pharma wants to buy, that’s what everyone wants to buy. At least for tech companies, the more, the larger the data set, the more well annotated it is, the more valuable it is. Data is the new oil…. in a small start-up company you have a lot more constrained resources. I think that you have to think intelligently and you’re less able to make mistakes.” (L1)

The neuro-entrepreneur above details how, despite their stated values, it is easier to sell data than ensure that the privacy of the end-user is protected. Neuro-entrepreneurs also shared how not sharing user data could slow the progress of innovation and hinder earning revenue. Those running smaller companies especially felt the obligation to focus on growth, which incentivizes leaders and entrepreneurs of these small companies to sell data and utilize end-user or patient data for profit. Participants also consistently noted that the consumer should own their data and had strong values toward transparency.

“I guess it [(misuse)] would be something that **goes against the expressed consent of the end user**. So, let’s imagine the end user is assuming whatever data is getting captured through a device or through a lab is going to be private and confidential and then somehow, the developer, or **someone else accesses that data and shares that in a way that is inappropriate**.” (C2)

“Patients should have the option as to who gets to see it [(the data)] or gets alerted.” (U5)

Many of the neuro-entrepreneurs reported that in order to mitigate harm to the end-user or patient, the end-user or patient should own their data, be aware of, and consent to all usage of that data, especially regarding third party usage. Other entrepreneurs noted the need to have data usage practices informed by a breadth of stakeholders.

“To protect the data, **I would need to listen to what the patient’s perspectives, what my team’s perspective and what leaders in the industry believe**. And through that, gather prominent insights and arrive at something that I believe would protect and preserve the data of our patients.” (D17)

Overall, the neuro-entrepreneurs are suggesting that end-users should control the fate of their own data and that this should be an implemented ethical practice, but again are not incentivized to do so at this point.

2. Access and justice: Neuro-innovations at their best can empower society, but the tech and insights are not always shared with everyone in society who might benefit.

Neuro-entrepreneurs state the importance of being aware of the implications of neurotechnology/neuro-innovation on macro and micro scales, especially regarding access.

“The fact that there’s so many people suffering from brain diseases and over one billion people diagnosed… in let’s say, the developed world. In many reports they say one out of three will develop a brain disease, I think that the impact is also pretty high. So, I’ve been very lucky to have that set of skills and knowledge to be able to work in this field…. It’s very important how we are going to regulate this and how are we going to use it in a way that **we don’t cause huge problems in the future where we have kind of like ‘the super elite’ and ‘the normal people,’** and that’s kind of abuse. And this is just one example.” (X8)

Another neuro-entrepreneur takes this future issue further, stating that neuro-innovation that is capable of human enhancement, particularly through neurotechnology, for example, can lead to discrimination that is even deeper than that which we have in society today.

“I mean, it can go a lot of scary ways. **It can go toward eugenics** and toward ranking people…” (E11)

Many participants noted that neuro-innovation has the potential to further divide society and further disenfranchise groups by creating and perpetuating prejudice and bias.

3. Neurodata and misuse: Current data regulations suffice but may not be sufficient for future implications and possible uses of brain data especially in the commercial space.

Misuse of neurodata was defined consistently as a lack of transparency and likely harm to the user or patient, which was suspected to be more likely in the commercial/non-clinical space by the participants. Most interviewees reported that current privacy and data regulations suffice due to the current limited clarity about future implications of brain data. In the future, however, neuro-entrepreneurs predict that it will be increasingly important to reassess the implications of brain data and monitor regulation and protection of user privacy.

“**I think in the future, it’s much more of a risk than now**. With that being said, I do think that it’s important for us to set a limit on privacy for brain data just so that people’s confidentiality is protected…. We should not limit brain data to just be data from the brain collected from an MRI, but we should also include any manifestation of behavior of the brain that could relate to a brain circuit and define underlying functionality.” (L1)

This neuro-entrepreneur discusses the difficulty in drawing bright lines of what constitutes brain data and that perhaps we should not. Due to this unclear categorization and these unknown future implications, our participants largely believe that brain data requires no extra enforcement than other types of biological data. However, the neuro-entrepreneurs also discuss how it is important to monitor brain data as technology progresses for future-proofing in order to prevent possible negative and harmful brain data usage in the future.

“I think right now, there’s still a lot of autonomy because these devices are non-invasive, they are at the will of the user. So, someone can put a device on, take the device off. There’s also the opportunity to choose to share data or choose not to share data, at least today, in today’s world. I think that in the future, once we get to fully invasive devices, **I think that autonomy will start to whittle away some more and once you start to surrender a lot of data for convenience**.” (V14)

4. Shifting societal norms: Unintended uses or access to data may lead to stigma, discrimination, power imbalances, and other unintended consequences, but the implications are not apparent to users or the entrepreneurs.

Many participants noted that neuro-innovation currently has and will continue to have the potential to change societal norms. As a vehicle for data creation and categorization, neuro-innovation will reshape society in some way, either major or minor (and positively or negatively).

“I think that this [the third party use of neuro-data] could impact insurance companies…. how to handle that data is I think really unclear at this point… interaction with your brain with technology will 100% redefine and change societal norms because **we’ll have to ask ourselves how we live with that technology** and how our brain relates to technology, how we want to move forward as a society with that.” (A7)

Neuro-entrepreneurs suggested that neuro-innovation will permeate different aspects of daily life on a global scale, whether through direct usage or even lack of access. Other entrepreneurs noted the possibility for shifting societal norms in a more positive way.

“I think demystifying the brain and translating discovery from brain investigations will make certain disorders less mysterious. And as they become less mysterious with the pathology or something reproducible about what is wrong, that will **reduce stigma as opposed to enhance stigma**.” (J15)

5. Autonomy: Neurotechnology/neuro-innovation can enhance or diminish autonomy.

Participants frequently noted how neurotechnology and neuro-innovation has the potential to influence how an individual operates in the world and can be a double-edged sword. The scale at which these effects might occur or could unfold in the future, will become apparent as the neurotech space grows and is adopted by a larger market.

“I do think, in the future, that trend will continue. Meaning, **brain tech will empower the autonomy and the decision making of people**. However, there will also be instances where there is abuse and when there are ways in which brain tech is used to reduce the autonomy of individuals. So, we have to anticipate those risks and know how to mitigate them.” (C2)

“People that are in a certain coma or something with EEG devices, you can already kind of capture certain brain patterns and you can already establish communication… of course, it depends if it can also pose the opposite, a huge restriction if. like science fiction movies type of thing like *Black Mirror* and reading minds and then, passing this information to those big corporations or governments… **so it can go both ways I think it can be very empowering, but also it can be limiting**.” (X8)

The neuro-entrepreneurs explain how neurotechnology can, for example, enhance the autonomy of patients whose life experiences may be viewed as limited due to disease or disability. The neuro-entrepreneurs also explain how technology can be abused. There may be instances when competitive advantage and efficiency are offered, yet this still could come at a personal cost for some members of society as illustrated by the quote below.

“So [(with neurotech)] you make things more and more efficient and you, as you **expand somebody’s ability to multitask and take on more complicated things, that causes personal stress**, right? And it increases the level of competitiveness and speed at which things are done.” (I3)

The above example also overlaps with the theme of changing societal norms, but the participant is referring to a loss of autonomy through social pressure to use technology to be competitive with a technologically enhanced workforce. Neuro-entrepreneurs are currently unclear as to which neuro-innovations could specifically be used to hinder autonomy but mention that any technology that is meant to enhance the experiences of the user can also be abused to hinder the ability of an individual.

#### III. How Would Ethics Fit With the Neuro-Innovation/Creative Process and/or the Context of Innovation?

**Table:** 

C. How would ethics fit with the neuro-innovation/creative process?
1. Ensuring/maximizing impact: Ethics is desired for preventing harm to the end-user and ensuring impact.
2. Nimble guidance vs. restriction: Ethics enforcement is viewed as restrictive and slowing, primarily through the lens of regulation; however, ethical guidelines (in any format) can be helpful tools throughout and after the innovation process.
3. Incentivization: Incentivization of ethical behavior is missing and desired.
4. ROI (return on investment) and growth opportunities: Ethics could help neuro-entrepreneurs to maximize the uses of their products while also mitigating negative uses.

1. Ensuring/maximizing impact: Ethics is desired for preventing harm to the end-user and ensuring impact.

Neuro-entrepreneurs noted that ethics was seen as a critical part of ensuring a positive impact on end-users and patients, possibly playing a role in the innovation process and facilitating discussions across stakeholder groups.

“I would like to see all those big groups **representing users and patients working with the neuro-entrepreneurs to help shape that innovation** in a way that is as useful as possible.” (C2)

Many participants held the view that in order to maximize impact within the current pace of technology, those who are impacted must be involved in the conversation. Participants pointed to a broad range of stakeholders.

“I think that **ethics should be an ongoing conversation between all the stakeholders**… so, the scientists, policy makers, government, investors who support those entrepreneurs, investors and advisors who help make companies grow, and in some cases I would say, the subset of the investor community that’s also philanthropic.” (F4)

In the above quote, the neuro-entrepreneur explains that patients and end-users should be included in the conglomerate of diverse stakeholders who contribute intellectually to an idea or innovation. While stakeholder engagement, particularly with end-users and patients, was frequently mentioned by neuro-entrepreneurs in our interviews, they rarely elaborated on the specific activities in which they currently are or have engaged end-users in the innovation pipeline discussion.

To ensure ethics and technology are evolving at similar paces, participants noted that ethics should be interwoven into the innovation process.

“My opinion is that **it [ethics] should really be infused in the culture of neuro-technological development,** because there are a lot of product development and technology development areas where the essence of who we are as people and what defines us is not necessarily touched upon.” (F20)

“It’s important for us **to reassess the pace of technology.**” (L1)

The participants mention how ethics in innovation must be an ongoing conversation amongst all diverse stakeholders to ensure holistic considerations and maximal impact. In order to maximize impact, participants noted that ethics also needs to be as nimble as the tech, keeping up with the science and offering opportunities to take stock throughout the innovation process. This sentiment also leads us to the next category.

2. Nimble guidance vs. restriction: Ethics enforcement is viewed as restrictive and slowing, primarily through the lens of regulation; however, ethical guidelines (in any format) can be helpful tools throughout and after the innovation process.

Some participants viewed ethics as a guide, a helpful tool for neuro-entrepreneurs throughout the innovation process for thinking outside of the box and promoting creativity. However, ethics, if seen as stringent regulation, enforcement, or law, was perceived as hindering the innovation process and perhaps not nimble enough.

“I think that ethics guides creating new things and creativity in a lot of ways…. I think ethics is important. **I also think that we can’t slow our pace of innovation.** There’s so much to know, to discover. I think it’s a compromise.” (L1)

“Regulatory **guidelines are based around existing technology**. So as technology advances, then there won’t be any regulatory guidelines for how to handle the new brain data that will be coming out.” (E11)

Participants generally describe how ethics must be guiding but not restrictive in order to be helpful for neuro-entrepreneurs. The participants noted that useful guidelines could be employed now and would provide guardrails that anticipate future ethical risks (future-proofing).

“I think that, so in the long term, when you actually have people that you have to protect and interests that you need to protect, there needs to be regulation, but in an early context where people are still creating the field, **having super heavy-handed regulation can stifle innovation**. And so, I think in the early stages, **having guidelines so that companies and organizations can start to explore these questions** because you really don’t know, and you can’t possibly think through all of the various permutations as to how this technology would play out and **so you’ve got to give people the opportunity to explore**.” (V14)

“I mean, guidelines would be something more universal that we could agree on as a culture of neurotechnology developers, and **I think that would be helpful so we’re not just all making it up on our own as we go**, … to **help us frame the risks** that we’re taking.” (F20)

3. Incentivization: Incentivization of ethical behavior is missing and desired.

Neuro-entrepreneurs are motivated by economic opportunities and the current state of the market, with ROI as a standard positive reinforcement and regulation seen as punitive. Participants suggested that shifting this model to incentivizing ethics could motivate entrepreneurs to innovate ethically.

“**I think clearer incentives to do the right thing**. and these can come in different ways…. They can come from more favorable regulation. It can come from those user groups, I mentioned them earlier. Maybe **rewarding good behavior**. For example, nothing prevents AARP or any of these big groups representing millions of patients from saying, we’re willing to review all the new neurotech in the field and whoever is the best one or two applications that really help people and that care about our privacy and that care about ethics, we’re going to help promote them among our users and help drive adoption. It could be **positive media coverage** to reward the people who take care of these things. So, those are the things that I think would be beneficial in this field, as in any other emerging field.” (C2)

This neuro-innovator details how incentives can motivate ethical behavior, and specifically how, for example, current positive reinforcers and recognition via mass media or positive press (that could lead to economic opportunities) could be integrated into the model for how ethical innovation practices can be maximized by neuro-entrepreneurs. Other entrepreneurs suggested other creative motivators like a kind of ethics tax for those who move into more questionably ethical areas.

“What is incentivized and what is not incentivized and I do think government regulation, through **incentives, can work much like tax**, carbon taxes. I do think such things could be useful for incenting data sale if you had a data pact, if it’s above a certain level of tiered privacy infringement.” (L1)

4. ROI and growth opportunities: Ethics could help neuro-entrepreneurs to maximize the uses of their products while also mitigating negative uses.

The entrepreneurs discussed how ROI is a central facet of business and how this impacts the direction of product innovation. In order to maximize profit, entrepreneurs need to maximize uses for their technology.

“Often when you’re an entrepreneur, **you need to also maximize the value for all the shareholders, which means that you need to make more and more revenue.** So, often it will make sense to go in different directions.” (X8)

By attempting to maximize impact and return on investment (ROI), while combating possible negative uses of neurotechnology, neuro-entrepreneurs can feel that they are put in a challenging position. Ethics and innovation can be viewed as pulling entrepreneurs in opposite directions, resulting in tension and conflict regarding innovation strategy.

However, many participants described the importance of exploring externalities and possible negative scenarios for outcomes of research and development.

“**You never know** whether there’s going to be some **application that could be potentially profitable or something that could be dangerous**.” (I3)

“**I don’t think you’re doing a very good job of being an entrepreneur if you’re not thinking about it [(multiple uses)]** that way. Because, A, if you’re not thinking about it that way, you’re not thinking about your exit opportunity and you’re not thinking about the externalities, just like, ‘Here’s all the ways that my product could have an effect.”’ (Y9)

Many participants also describe how ethics is often engaged to think out of the box and enhance creative decision-making especially when looking for growth and expansion opportunities.

“**Ethics should not be compromised in any way and to innovate**, you have got to think out of the box. So they’re connected.” (X8)

“I would say that, again, the relationship lies where both creativity and innovation are applied. That’s where ethics really comes in…But, you know, on the other end of things, I think where it comes into play in a practical sense, beyond just informing your every, waking decision, is where, for example, **a scientist is going to determine which among the probably, tens to hundreds of ideas that they have, which ones that they pursue and seek grant money for**.” (F4)

## Discussion

### Primary Findings

We aimed to understand the values that motivated neuro-entrepreneurs, the ethical tensions in their work, and whether they viewed ethics and innovation as compatible, mutually exclusive, or perhaps somewhere in between.

The qualitative data collected from these neuro-entrepreneurs is a preliminary exploration of their attitudes and needs for (neuro)ethics, but we believe is an important entry into a gap area that warrants deeper discussion. One neuro-entrepreneur noted that this study is timely:

“in innovation and the consumer space, the genetic stuff [ethics conversation] is already widespread, but the neuro stuff is not quite there yet.” (O6)

The results of the qualitative interviews from this study suggest that neuro-entrepreneurs are beginning to recognize key ethical questions throughout the innovation process and forecast future ethical issues with the continued widespread use of neurotechnology. In our interviews, these concerns clustered into five areas: Data Ownership, Access and Justice, Neurodata and Misuse, Shifting Societal Norms, and Autonomy, pointing to overlap with all key questions listed in the NeQN.

In our study, many neuro-entrepreneurs had consistent values motivating their work. These included broadly benefiting and advancing humanity through empowering individuals in both clinical and non-clinical contexts. Their key values came in conflict with realities of the innovation process around data acquisition and sharing/selling, dissemination, distribution and repurposing of tech in society particularly from clinical to non-clinical domains. The interviews also surfaced a consistent interest in engaging a broader range of stakeholders, particularly end-users, yet few innovators in our study elaborated on any strategies or existing practices of diverse stakeholder engagement.

In our study, entrepreneurs could envision ways for ethics to be useful and integrated into the neuro-innovation enterprise by helping to future-proof with foresight and risk mitigation.

Necessary prerequisites for neuroethics integration would be the introduction of incentivization structures and ethics and guidance keeping pace with the science (staying nimble),–something participants did not feel legal regulations could do. Incentivization structures were described as being motivating, in that they would publicly acknowledge ethical innovators or relief from a kind of “ethics tax” for those not meeting an ethical standard.

### Needs for Practical Guidance

Many neuro-entrepreneurs who participated in this study indicated that ethics could be a useful component of the innovation process. Integrating ethics within the beginning (ideation, prototyping, testing) stages of the innovation process and at the deployment stage ensures end-user and patient safety and assists the entrepreneur in deciding which projects are viable and beneficial for humanity. Integrating ethics in the beginning phases can also ensure that ethics is not just used as a tool to perform damage-control on consequences of harmful neurotechnology – but as a component of the neuro-innovation process itself (future proofing the innovations and tech).

In order to incorporate ethics into the process of innovation from start to finish, we need to more deeply understand the roadblocks and bottlenecks that prevent its integration. In these interviews, we noted that neuro-entrepreneurs need a compelling business case for neuroethics and incentives for aligning their work with attention to neuroethical concerns. In many ways, the discussions of ethics sometimes veered to far-future scenarios and felt a bit separate from the innovation process. We suspect neuro-entrepreneurs would benefit from a neuroethics strategy and toolkit to help them integrate neuroethics into their work. Many pointed to critical neuroethical issues highlighted in the NeQN, but neuro-entrepreneurs likely need a simplified, more readily accessible rubric than the elaborated questions offered by the NeQN.

The neuro-entrepreneurs also articulated concerns that restrictive laws or enforcement would hinder innovation and project goals. However, the entrepreneurs were open to guidance that was nimble enough to keep up with their evolving science.

To be clear, there have been some ethical guidelines and principles for innovators. For example, in 2014, the UNICEF Innovation Unit set out four ethical guidelines for its framework in global innovation, as well as principles for innovation:

“Innovation is humanistic: solving big problems through human ingenuity, imagination and entrepreneurialism that can come from anywhere.”

“Innovation is non-hierarchical: drawing ideas from many different sources and incubating small, agile teams to test and iterate on them with user feedback.”

“Innovation is participatory: designing with (not for) real people.”

“Innovation is sustainable: building skills even if most individual endeavors will ultimately fail in their societal goals.”

(Fabian and Fabricant, 2014).

While many of these points would likely resonate with our participants, the above points do not address the specific *neuroethical* themes we were exploring such as neural privacy, autonomy, or specific guidance on stakeholder involvement on thorny neuroethics issues. Notably, the Organization for Economic Cooperation and Development, a transnational policy and governance institute, identified neurotechnology as a singular technology that calls for a set of Neuroethics Principles. There have also been over twenty sets of principles and guidelines from collaborative efforts of scientists https://instituteofneuroethics.org/nx-guidance, policy makers and ethicists, but most struggle to be recognized and utilized in both the academic and industry sectors alike.

For many in neuro-industry, the stakes of ethical mishaps within neuro-innovation are applied on a larger scale, fraught with unknowns, and often disseminated in a more public domain (in clinics or consumer populations). We have an opportunity to co-create with more representative voices of the neuro-innovator community and end-user/patient communities to develop some practical rubrics, tools, and strategies that could robustly facilitate neuroethics integration into the neuro-innovation process. We see this project and these conversations within neuro-industry as a starting point and launching pad for developing practical tools to integrate neuroethics within neuro-industry and in the spaces between academia and the private sector.

### Limitations

While there are promising data from this study, there are some significant limitations. First, some neuro-entrepreneurs were largely recruited from one of the team member’s personal networks (Dr. Karen Rommelfanger). However, not all participants were aware of what neuroethics was or were familiar with neuroethics literature. Some participants had already implemented neuroethics within their efforts while some asked for clarification about the definition of neuroethics and its scope and impact on neuroscience thus far. As with qualitative studies, these results are not meant to be generalizable and are meant instead to capture a breadth and depth of themes. We are planning a future quantitative survey-based study to capture perspectives from a broader and more diverse population across several dimensions such as gender, racial identities and geographic location. A follow-up exploration of the unique and overlapping neuroethics needs and barriers between academic and private-sector researchers could also provide insights for smoother collaboration and cohesive strategies for creating more societally impactful neuroscience.

## Neuroethics, Theories of Innovation and Creativity

At its core, neuroethics is a field that interrogates and addresses value conflicts in neuroscience with an intentional and careful exploration of multiple perspectives of diverse stakeholders. Some theories of innovation, such as the Cultural Swirl Theory, similarly describe innovation as a process that requires bringing together a mix of skilled and diverse stakeholders who can collectively bring about cultural and/or technological innovation and change. Under the Responsible Research and Innovation (RRI) framework, the importance of multi-stakeholder engagement is also considered a necessity to ensure multiple voices contribute to determining the ethical directions for innovation (RRI-Practice, 2017). With RRI, innovators are also asked to be willing to critique their own actions and systems of thinking in order to comprehensively analyze the risks and benefits of research and innovation. This ability to critically analyze how one’s individual processes affect the collective work of the group is called “reflexivity,” (Stilgoe et al., 2013). So, creating social good can be enabled by not only exploring diverse perspectives, but also by critically analyzing our own actions and systems of thoughts in a reflexive way. Reflexivity and self-awareness on both an individual and organizational level can guide the innovation and research process in a socially responsible and ethically sustainable way (Lubberink et al., 2017).

Upon first glance, it may seem that the fast pace of innovation may be at odds with the additional time that addressing ethical considerations might require. However, studies have demonstrated that creativity and ethics are complementary and equally important to innovation (Bierly et al., 2009). Buchholz and Rosenthal (2005) further discuss how the spirit of entrepreneurship can align with ethics, demonstrating how, in order to thrive as an entrepreneur, one needs a strong moral decision-making toolkit that can be readily accessed for fast-paced decision-making. They discuss the shortcomings of traditional top-down ethical approaches such as deontology and virtue ethics in modern decision-making and introduce the concepts of moral imagination and moral sensitivity as part of a new bottom-up ethical model termed “process ethics.” In process ethics, the decision-making activity or process that leads to the final outcome of ethical-decision-making is critical and benefits from the ability to have awareness (i.e., moral sensitivity) of and an ability to envision a full range of ethical possibilities and consequences (i.e., moral imagination). According to Minnette Johnson and Patrick Murphy, moral imagination is the ability and willingness to blend both creativity and ethical thinking (Austin, Texas: McCombs School of Business, University of Texas, 2018). Mark Johnson, philosopher and author of *Moral imagination*, defines moral imagination as the ability to envision a multitude of possibilities regarding the future in order to solve ethical challenges that result from specific actions (Johnson, 1993). Entrepreneurs can achieve these quick creative and moral decisions by sharpening their moral sensitivity and imagination as part of their innovation toolkits. Although not explicitly stated, our participants alluded to the importance of moral imagination in the process of considering how to maximize the impact of their innovation and for reimagining growth opportunities and future uses for their neurotech.

Bierly et al.’s (2009) research indicates that the creative process is correlated to an individual’s ethical framework. Through a sampling of business students, the researchers found a positive correlation between creativity and ethical relativism. Ethical realism is defined as divergent thinking from universal moral principles and in general is critical of notions of moral universality. These data suggest that creative individuals are less likely to conform to universal morality and be open to a plurality of value frameworks. The same study found a positive correlation between creativity and ethical idealism, which is defined as a gauge of one’s concern for the welfare of others and the belief that harming others is always avoidable.

**FIGURE 2:**
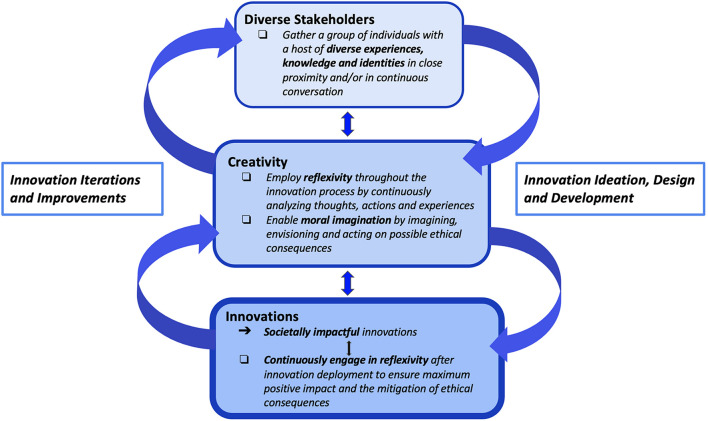
Some theories of innovation, such as the Cultural Swirl Theory, describe innovation as a process that requires bringing together a mix of skilled and diverse stakeholders who can collectively bring about cultural and/or technological innovation and change. Ideally those diverse stakeholders bring about a cognitive diversity. This cognitive diversity enhances an ability to incorporate and consider multiple perspectives. Further, creativity is enhanced by a practice of reflexive exploration and moral sensitivity to unspoken values and an enhanced moral imagination for societally impactful innovations.

Likewise, Cultural Swirl Theory also suggests that having a diverse set of stakeholders and access to a plurality of perspectives would help sharpen this moral imagination and ability to engage in more reflexive ethical awareness. Here, we note that one of the key features of an ethics and neuroethics strategy and/or toolkit is being able to examine a problem from multiple perspectives as framed by the Cultural Swirl Theory (engaging in multiple perspective-taking to enrich neuro-innovation with moral imagination as offered by a neuroethics framing) (Figure 2).

Taken together, this theoretical mapping and prior research on ethics and innovation suggest that a reasonable hypothesis to further explore with this work is that **neuroethics could be a critical component and even advance the neuro-innovation and neuro-industry enterprise**.

## Future Directions

From our preliminary study using the framing of the Cultural Swirl Theory and process ethics in innovation related to moral imagination and sensitivity, we suggest that **neuroethics is not contrary to, but instead can enrich neuro-innovation.**

The results also suggest that neuroethics can be compatible and thoughtfully integrated into the neuro-innovation process given that specific conditions are met – such as promoting incentives for engaging neuroethics topics, ensuring that the neuroethics keeps pace with the science, and demonstrating a clearer value proposition. Our preliminary research indicates that both academic and government-based neuroscience research and neuro-industry would benefit from neuroethics tools and actionable steps and that there is general interest in the neuro-industry community for utilizing a lens of neuroethical inquiry in the innovation process. More work will be needed to understand the extent of specific ethical concerns across the broader neuro-industry community and the processural roadblocks that prevent neuroethics integration in the neuro-innovation process. Future collaborative research will aim at developing concrete action items and an actionable roadmap for neuroethics integration into neuro-innovation. **With the rapid growth of a relatively young neuro-industry community, the time is ripe to more proactively and robustly pursue a collaborative effort between academic science and industry to engage and align around neuroethics for more societally powerful neuro-innovations.**

## Data Availability Statement

The raw data supporting the conclusions of this article will be made available by the authors, without undue reservation.

## Ethics Statement

The studies involving human participants were reviewed and approved by Emory University IRB, by assessing the perceived value of neuroethics questions and policy to neuro-entrepreneurs. Written informed consent for participation was not required for this study in accordance with the national legislation and the institutional requirements.

## Author Contributions

AUM and KSR contributed to conception and design of the study. All authors contributed to data analysis. AUM wrote the first draft of the manuscript. AUM and KSR wrote sections of the manuscript. All authors contributed to manuscript revision, read, and approved the submitted version.

## Conflict of Interest

KSR offers neuroethics consultation for nonprofits and startups but did not offer consultation to any participants of this study. The remaining authors declare that the research was conducted in the absence of any commercial or financial relationships that could be construed as a potential conflict of interest.

## Publisher’s Note

All claims expressed in this article are solely those of the authors and do not necessarily represent those of their affiliated organizations, or those of the publisher, the editors and the reviewers. Any product that may be evaluated in this article, or claim that may be made by its manufacturer, is not guaranteed or endorsed by the publisher.
