# Supplementary material for: Assessing the Perceived Value of Neuroethics Questions and Policy to Neuro-Entrepreneurs
Source: Front Neurosci. 2021 Oct 13;15:702019. doi: 10.3389/fnins.2021.702019 (PMC8548819; doi:10.3389/fnins.2021.702019)
Supplement: Supplementary file 1 [file Data_Sheet_1.docx]

**Appendix: Participant Demographics**

Location:
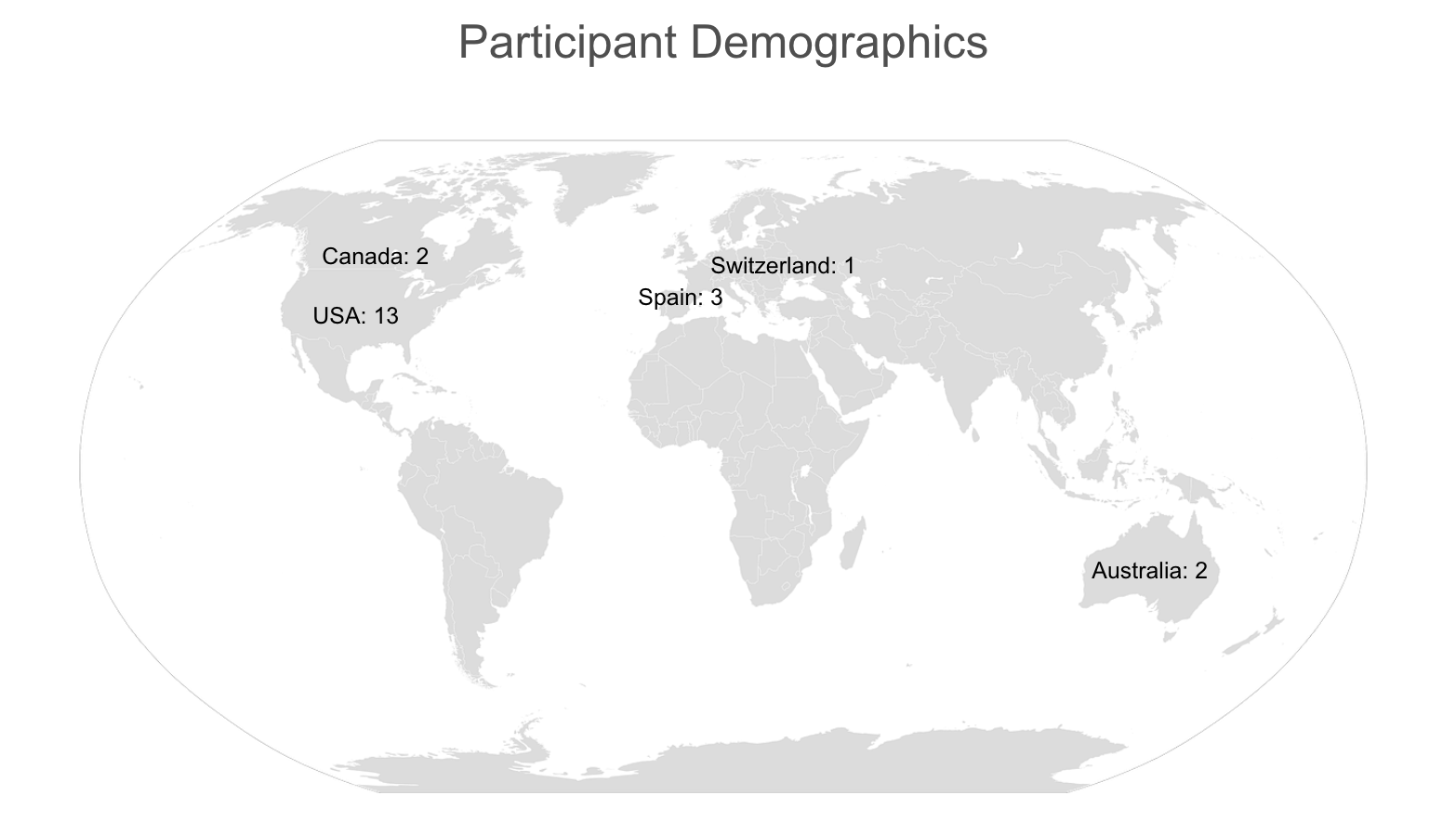

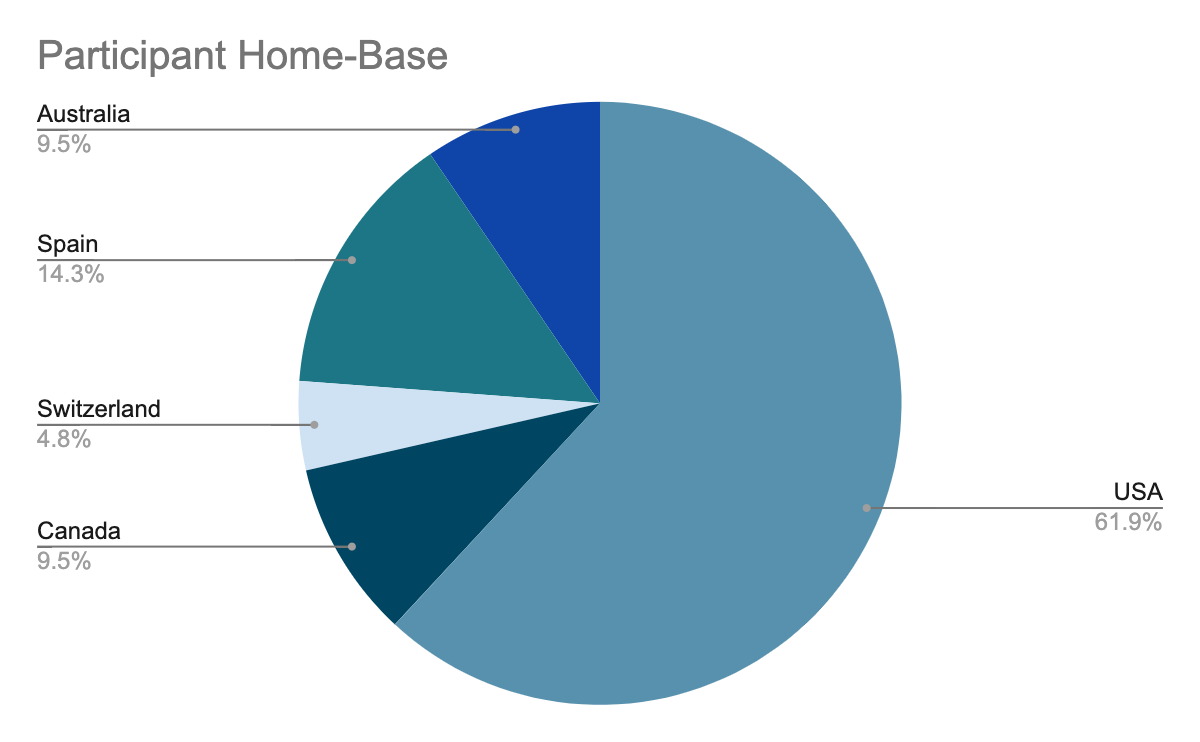


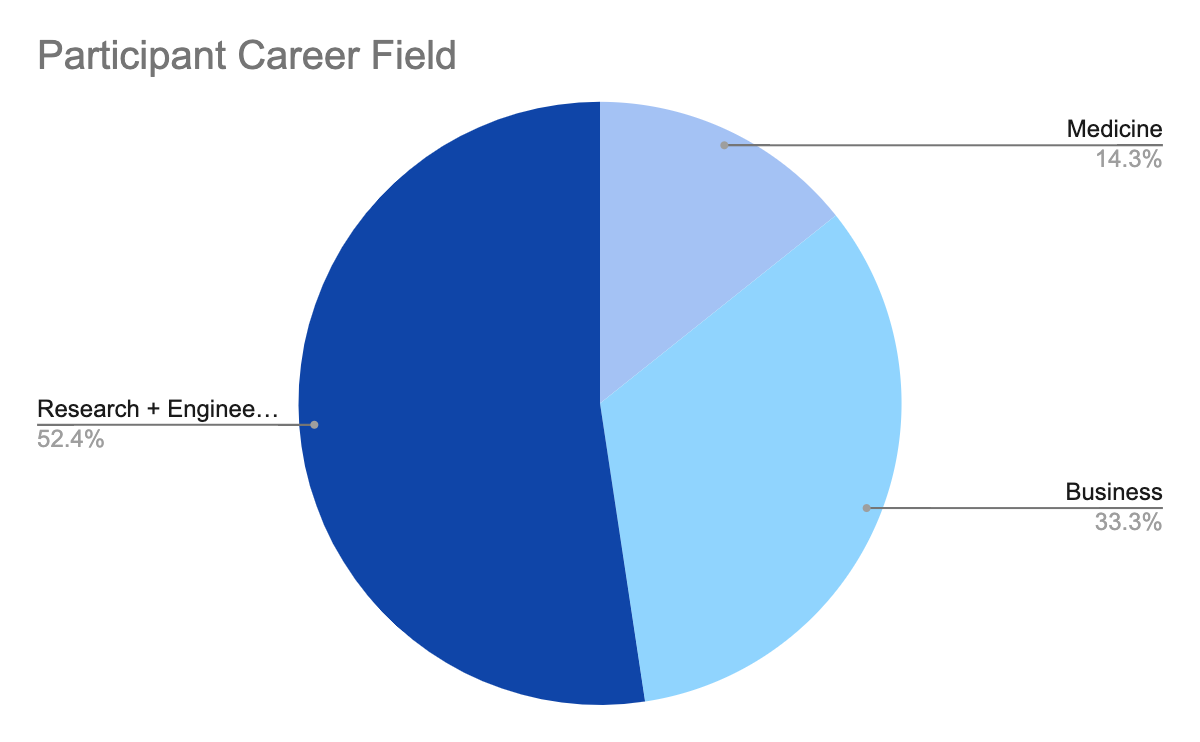


| **Gender** | Male | Female |
| --- | --- | --- |
|  | 13 | 8 |

| **Highest Level of Education** | **overlap with MBA* |
| --- | --- |
| MBA | 6 |
| PhD | 10 |
| Bachelor | 3 |
| MD | 3 |
| Other Masters | 2 |
